# Staphylococcus aureus Orbital Abscess With Impending Compressive Optic Neuropathy in an Immunocompetent Individual With Subclinical Bacteriuria: A Case Report

**DOI:** 10.7759/cureus.50693

**Published:** 2023-12-17

**Authors:** Amirul Hasbi, Ismail Shatriah, Haslinda A Rahim, Akmal Haliza Zamli, Evelyn Tai

**Affiliations:** 1 Department of Ophthalmology and Visual Sciences, School of Medical Sciences Universiti Sains Malaysia, Kubang Kerian, MYS; 2 Department of Ophthalmology, Hospital Raja Perempuan Zainab II, Kota Bharu, MYS; 3 Department of Ophthalmology, Hospital Tengku Ampuan Afzan, Kuantan, MYS

**Keywords:** urine culture, cutaneous incision and drainage, immunocompetent, staphylococcus aureus, orbital abscess

## Abstract

This is a case of an orbital abscess evidenced radiologically in a 41-year-old female with no comorbidities. She was healthy and had no history of trauma or infection of the adjacent structures. She denied having symptoms of upper or lower respiratory and urinary tract infections. The decision for surgical drainage was made following a slow response to antimicrobial agents after 24 hours, a progressive painful erythematous eyelid swelling, and further deterioration of vision. Her clinical condition and visual acuity improved following cutaneous incision and drainage. Culture and sensitivity results for urine and orbital abscess were positive for Staphylococcus (S.) aureus. The patient regained full visual recovery without any sequelae. In conclusion, an orbital abscess is a blinding and life-threatening condition that rarely occurs in immunocompetent individuals and uncommonly arises from distant sources. A high index of suspicion, early institution of appropriate diagnostic imaging, and aggressive medical and surgical treatment are necessary for a favorable visual outcome in orbital abscess cases.

## Introduction

An orbital abscess is an extension of a periorbital infection that is commonly seen in immunocompromised individuals, and it can be potentially sight-threatening or even life-threatening [1]. Oftentimes, the infections originate adjacently, and the most common site is the paranasal sinuses, predominantly the ethmoidal and maxillary sinuses [2,3].

Orbital abscesses can result in irreversible blindness via compressive optic neuropathy. The increasing intra-orbital pressure created by the abscess causes compartment syndrome, as the orbit is a closed compartment with limited ability to expand, leading to substantial optic nerve compression that can result in permanent loss of vision. The infection and inflammation can also directly damage the nerve. Extension of the infection may result in the formation of cavernous sinus thrombosis and its sequelae, which can lead to death.

The immune status of an individual is highly correlated with the acquisition of staphylococcal infection [4-6]. Studies have shown that patients in an immunocompromised state are likelier to acquire the infection than immunocompetent individuals [4-6]. This study highlights a rare case of a Staphylococcus (S.) aureus orbital abscess with impending compressive optic neuropathy in a healthy immunocompetent adult with subclinical bacteriuria who was treated successfully with antibiotics and cutaneous incision and drainage.

## Case presentation

A 41-year-old female with no comorbidity was referred to the ophthalmology team complaining of redness and worsening swelling of the left upper and lower lids over two days. This was accompanied by a severe throbbing pain. The patient denied any recent ocular trauma, insect bite, nasal discharge, or dental problems. She had no history of upper or lower respiratory tract or urinary tract infections.

During the initial presentation, the patient was comfortable and normotensive with a normal heart rate. Her body temperature was 38°C, and she had normal capillary blood sugar.

**Figure 1:**
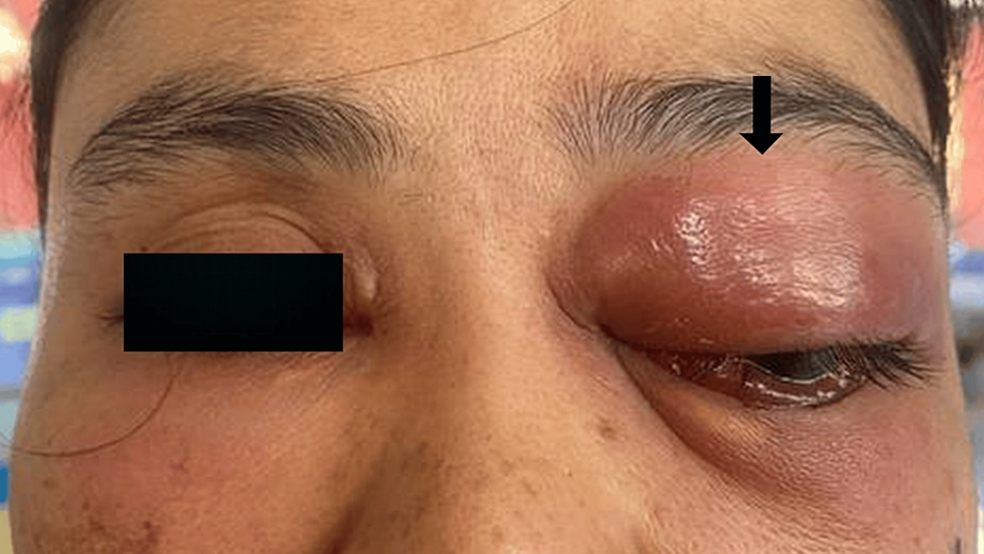
The patient's left eye at initial presentation The left upper and lower eyelids were swollen and erythematous (arrow).

Ocular examination revealed that visual acuity was 6/6 (20/20) in both eyes. The lid was swollen, with diffuse tense and tender periorbital swelling and erythema of the overlying skin extending to the left cheek. The eye was proptosed (8 mm) with a lagophthalmos of 6 mm (Figure 1). Elevation was restricted in the left eye. There was no punctum or bite mark seen on or around the eyelid. A corneal abrasion was noted inferiorly. There was no anterior chamber inflammatory reaction. The patient’s intraocular pressure was normal. The funduscopy examination was normal. The optic nerve function tests of the left eye were unremarkable, except for a slight reduction in red saturation. The right eye examination was unremarkable.

**Figure 2:**
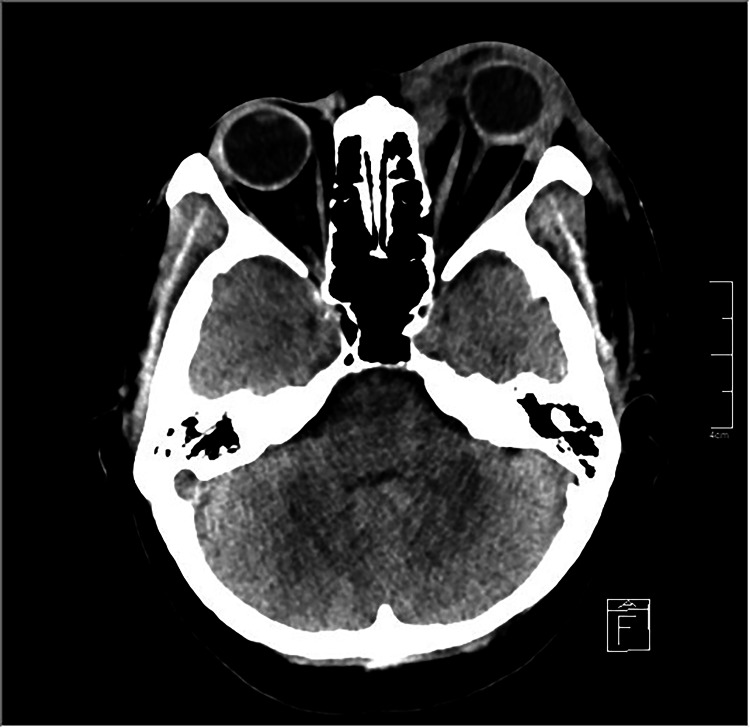
Plain CT image of the orbit, axial view The left eye appears proptosed with the presence of an enhancing collection in the left orbit located medially in the extraconal space. The preseptal soft tissue is thickened with the presence of fat streakiness. The globe and its content are preserved. The ethmoidal sinus is clear, and no enhancing focal brain parenchymal lesion is seen.

An urgent computed tomography of the orbit and paranasal sinuses revealed a pus collection in the left orbit located medially in the extraconal space, extending from the superomedial area to the inferomedial area (Figure 2). The ethmoid, maxillary, and sphenoid sinuses were clear. The white blood cell count was 17.5 × 109/L and 80% neutrophil predominant. Urine microscopic examination showed the presence of leucocytes and nitrates.

Based on the clinical and radiological findings, the patient was diagnosed with a left orbital abscess. She was started on 1 gram of intravenous ceftriaxone daily, 500 mg of intravenous metronidazole every eight hours, topical moxifloxacin 0.5% one drop hourly, topical chloramphenicol ointment 1% every 8 hours, and a regular warm compression. Assessments of extraocular motility, visual acuity, relative afferent pupillary defect, light brightness, red saturation, and confrontation test, were performed on a regular basis.

**Figure 3:**
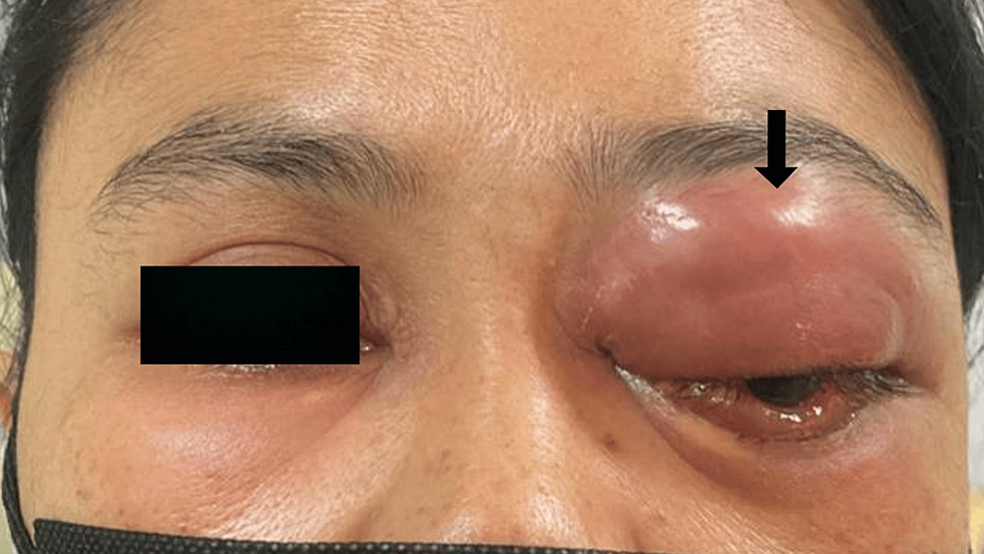
Left eye prior to surgical cutaneous incision and drainage of the orbital abscess. The worsening condition of the left eyelid swelling (arrow) despite being on intravenous antibiotics.

The following day, the patient’s visual acuity deteriorated to 3/60 (20/400) pinhole 6/60 (20/200) with worsening painful erythematous eyelid swelling without detectable relative afferent pupillary defect (Figure 3). Light brightness and red saturation were affected, and she developed complete ophthalmoplegia. The eyelid swelling became diffusely enlarged with the presence of a pus point on the swelling. The intraocular pressure of the left eye raised to 25 mmHg hence topical timolol 0.25% twice daily was started. 

In response to evidence indicating impending compressive optic neuropathy, a cutaneous incision and drainage procedure was performed. Five mL of purulent material was aspirated. The culture revealed S. aureus, which was sensitive to the current antibiotic administered. Urine culture revealed the same pathogen, which was sensitive to similar antibiotics. However, no microorganism was isolated from the blood culture. The blood sugar and renal function tests were within normal ranges.

**Figure 4:**
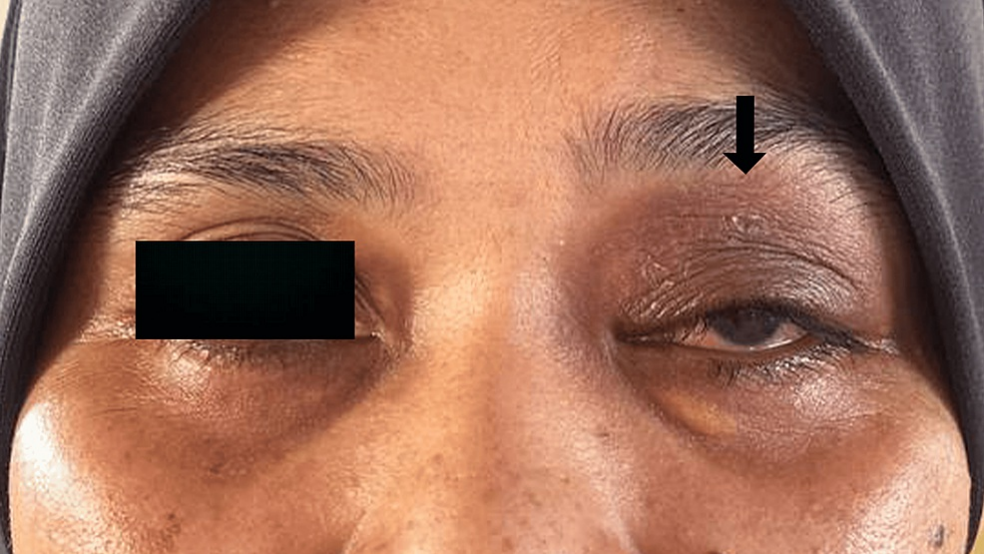
The patient's left eye after the surgical procedure and completion of 14 days of antibiotics. The eyelid swelling and erythema subsided (arrow).

Left visual acuity improved to 6/6 (20/20) after the administration of 14 days of intravenous antibiotics (Figure 4). The eyelid swelling subsided, and she was discharged from the hospital. Serial outpatient visits showed no recurrence after four months.

## Discussion

An orbital abscess is a serious emergency characterized by the extension of periorbital infection intra-orbitally and evidenced by a collection of pus in the orbital fat, which can either occur inside or outside the muscle cone. Diabetes mellitus is the most common risk factor for developing orbital and ocular infections [7]. It is believed that hyperglycemia in diabetes impairs the immune response, leading to an inability to curb the spread of invading pathogens. As a result, people with diabetes are known to be more prone to infections, as they are immunocompromised [8].

Bacteria are the most common pathogenic cause of orbital cellulitis, and the most common bacteria are the Staphylococcus, Streptococcus, and Pseudomonas species. Occasionally, the infective pathogen remains unknown despite culture sampling and isolation efforts [9,10]. The well-known species of Staphylococci, which are Staphylococcus aureus and Staphylococcus epidermidis are considered opportunistic pathogens [6]. Studies on adult populations have revealed that immunosuppression is linked to an increased risk of Staphylococcus aureus colonization compared to immunocompetent individuals [4-6]. It is noteworthy to emphasize that the patient exhibited immunocompetence and contracted a Staphylococcus aureus infection. This occurrence may be attributed to the virulence and aggressiveness of the organism, potentially leading to a more severe manifestation of the infection, even in individuals with a competent immune system [11].

Early commencement of the systemic antibiotic prior to presentation yielded a negative blood culture result consistent with other reported cases [3,12]. Infections in the orbital abscess predominantly arise from the adjacent structures, as mentioned in reported cases summarised by Zawadzki et al. [9]. The presentation of the patient in this case study was slightly misleading, as she did not exhibit signs of infection pertaining to the adjacent structures but instead had asymptomatic bacteriuria with the isolation of Staphylococcus aureus in the urine. The pus from the orbital abscess confirmed the growth of Staphylococcus aureus as the offending organism. Staphylococcus aureus is an uncommon cause of urinary tract infection, with a prevalence between 0.15% and 4.3%; nonetheless, it was the source of infection in this patient [13].

The rapid progression of the disease warrants prompt management, often with a combination of antimicrobial therapy and surgical drainage [14]. It is vital for the clinician to identify the signs of compressive optic neuropathy, and if present, urgent surgical drainage is mandatory [2-3,9,14]. Prompt surgical drainage may prevent vision loss. The common surgical drainage methods are external orbitotomy and endoscopic drainage. However, in this patient, cutaneous incision and drainage proved sufficient for draining the pus. This method has also been performed in orbital abscess cases, as reported by Zawadzki et al. [9].

The cutaneous incision and drainage method helps lower the intra-orbital pressure by creating an exit point for the pus and subsequently relieving optic nerve compression, thus preserving vision. It was suitable in this case because the abscess had progressed and extended more anteriorly and was located superficially and close to the skin, as evidenced radiologically. This method can possibly leave a facial scar (as it would in orbitotomy), but the recovery is postulated to be more rapid without any profound complications once the abscess is fully drained, as compared to the external orbitotomy [9].

## Conclusions

Immunocompetent individuals remain susceptible to the development of a severe Staphylococcal infection due to the virulence factors of the bacteria. A staphylococcal orbital abscess is a severe form of orbital infection with bacteriuria that can be a potential etiological source. Close monitoring of the ocular and systemic condition is mandatory to prevent sight-threatening and life-threatening complications. A high index of suspicion, early institution of appropriate diagnostic imaging, and aggressive medical and surgical treatment are necessary for a favorable visual outcome in orbital abscess cases.
